# 

**Published:** 1886-12

**Authors:** 


CONTENTS:

ARTICLE I—Post Graduate Study. By J. D. Moody, D. D. S............ 337
" II—Accuracy as a Condition of Scientific Progress. By J. Smith Dodge, Jr., M. D , D. D. S ........... 350
" III—Chemistry. By Charles L. Steel, M. D., D. D. S............ 366
" IV—Kava, Kawa, Kava-Kava—A Powerful Anæsthetic and Spinal Depressant ........................ 372

EDITORIAL, ETC.

Œsophagotomy for the Removal of an Artificial Denture .... .............. 376
Mouth-Breathing...... .................................................. 377
A New Substance.......................................................... 378

MONTHLY SUMMARY.

Pin-Hole Cavities and "Caries Interna."............ .......................... 378
Preventive Dentistry..................... .................................. 379
Power of an Operator over a Patient under the Influence of Nitrous Oxide 381
Hydronaphthol—A New Antiseptic ...................... .... ............... 382
A Curious Christmas Box.................................................. 384

